# The Insufficiency Intake of Dietary Micronutrients Associated with Malnutrition-Inflammation Score in Hemodialysis Population

**DOI:** 10.1371/journal.pone.0066841

**Published:** 2013-06-25

**Authors:** Jie Chen, Hongquan Peng, Kun Zhang, Long Xiao, Zhimin Yuan, Jianping Chen, Zhiyu Wang, Jingfeng Wang, Hui Huang

**Affiliations:** 1 Guangdong Province Key Laboratory of Arrhythmia and Electrophysiology, Guangzhou, Guangdong Province, China; 2 Department of Cardiology, Sun Yat-sen Memorial Hospital of Sun Yat-sen University, Guangzhou, Guangdong Province, China; 3 Department of Radiation Oncology, Sun Yat-sen Memorial Hospital of Sun Yat-sen University, Guangzhou, Guangdong Province, China; 4 Renal Division, Kiang Wu Hospital, Macau SAR, China; 5 Department of Blood Purification of the Second Affiliated Hospital of Guangzhou Medical College, Guangzhou, Guangdong Province, China; 6 Department of Nutrition, Sun Yat-sen Memorial Hospital of Sun Yat-sen University, Guangzhou, Guangdong Province, China; 7 School of Chinese Medicine, The University of Hong Kong, Hong Kong, China; Universidade de São Paulo, Brazil

## Abstract

The relations between dietary micronutrient, nutritional status and inflammation in hemodialysis patients are still unclear. A cross-sectional study was performed in hemodialysis population. 75 hemodialysis patients from South China participated in the dietary and nutritional assessment. Clinical and dietary data were collected. Nutritional status was assessed by Malnutrition-Inflammation Score (MIS) in addition to related anthropometric measurements. And according to the MIS score, the whole hemodialysis patients were divided into normal nutrition group and malnutrition group. The results showed that mid arm circumference (MAC) negatively correlated with MIS (r = −0.425; *P* = 0.002). The area under the ROC curve (AUC) for MAC was 0.737 (0.614–0.859). Comparing with the normal nutritional group, lower dietary selenium (Se), copper (Cu), iodine (I) and manganese (Mn) intake were observed among patients with malnutrition (*P*<0.05). While no significant differences of diverse vitamins were found. In conclusion, MAC was effective indicator for assessing nutritional and inflammatory status (*P*<0.05). The reduction of dietary Se, Cu, I and Mn intake level may be alarming markers for malnutrition and inflammatory status in hemodialysis patients.

## Introduction

Malnutrition is very popular in hemodialysis patients, and is related with increased risk of morbidity and mortality [1]–[3]. Therefore, dietary status assessment is meaningful for those patients at risk with malnutrition. Inadequate dietary nutrients intake is an important cause for malnutrition. As we all know, protein, energy, even vitamins and trace elements intake are inadequate in most hemodialysis patients [4]–[6]. And the remarkable reduction of daily nutrients intake has been shown to be an independent determinant of reversible impairment of nutritional status [7]–[9]. Currently, we are still not clear about the accurate dietary micronutrients status, especially the trace elements of Chinese hemodialysis patients.

Assessment of nutritional status can be attained by anthropometry, subjective global assessment (SGA), Malnutrition-Inflammation Score (MIS), etc. SGA was firstly reported by Detsky [10], and is a valid method for assessing the nutritional status. MIS scoring system, a quantitative assessment method based on SGA, is another comprehensive scoring systems predicting morbidity and mortality as well as measuring nutritional and inflammatory status in hemodialysis patients [11], [12]. Malnutrition and inflammation often co-exist in hemodialysis patients, and are associated with accelerated atherosclerosis which is called malnutrition-inflammation-atherosclerosis syndrome [13]–[16]. This condition negatively affects the clinical outcome of hemodialysis population and increases the risk of cardiovascular events. It is well known that protein-energy wasting is associated with malnutrition. However, it is not the only form deciding malnutrition which is often accompanied by the deficiency of some micronutrients, such as zinc (Zn), selenium (Se), etc [6]. Several factors, such as dietary intake restrictions, gastrointestinal disorders, insufficient dialysis, and constant concomitant illnesses are responsible for inadequate dietary micronutrients intake among hemodialysis patients. However, until now there is scarcity of studies in indexed literature about the dietary micronutrients intakes in hemodialysis population in China. So, in this study, we investigated the dietary micronutrients intake and assessed the nutritional and inflammatory status by MIS scores in hemodialysis patients. And we also focused on the roles of micronutrients in nutritional status among hemodialysis population and assessed the relationship between dietary micronutrients status and nutritional and inflammatory status. The present study aimed at improving clinical outcomes in the hemodialysis patients.

## Methods

This study protocol conformed to the ethical guidelines of the 1975 Declaration of Helsinki as reflected in a priori approval by the Ethics Committee of Sun Yat-sen University. Patients included in this study were from 4 teaching hospitals in South China (Sun Yat-sen Memorial Hospital; the Second Affiliated Hospital of Guangzhou Medical College; Kiang Wu Hospital; Affiliated hospitial of School of Chinese Medicine, The University of Hong Kong). Written Informed consent was obtained from each participant and their medical records were studied by anonymous means.

### Study population

This is a cross-sectional, multicenter study to evaluate the actual micronutrients intake in hemodialysis patients. Seventy-five randomly selected hemodialysis patients participated in the dietary micronutrients and nutritional assessment. 27 patients were from Sun Yat-sen Memorial Hospital; 20 patients were from the Second Affiliated Hospital of Guangzhou Medical college; 13 patients were from Kiang Wu Hospital; 15 patients were from Affiliated hospitial of School of Chinese Medicine, The University of Hong Kong. Each patient had been undergoing hemodialysis for at least three months. Patients' demographics and duration of hemodialysis were attained by a detailed history from the patients and their cases records excluding severe infection and tumor, severe cardiac failure or respiratory insufficiency, dementia, psychiatric or neurologic diseases. Hospitalization within the last three months was also considered exclusion criteria.

### Anthropometric measurements

Several anthropometric measurements were done after termination of the hemodialysis sessions. Measurement of MAC and mid arm muscle circumference (MAMC) on non-access arm was used to evaluate the muscle mass. MAMC was calculated by the formula: MAMC = MAC- (3.1415× TSF) (TSF, triceps skinfold thickness). Body dry weight was measured between 10 and 20 minutes after termination of the hemodialysis sessions. All the measurements were performed 3 times. The body mass index (BMI) was calculated as the ratio between body dry weight in kg and the square of height in meters (kg/m^2^).

### Dietary micronutrients intake assessment

A three-day diet diary record assessed by well-trained dietitians was used to estimate the daily micronutrients dietary intake [11]. Dietary intake was recalled over the last hemodialysis treatment day of the week and the two subsequent non-dialysis days. The dietitians made the changes and corrections on the food record and used the Minnesota Nutrient Data System software (version 2005; Nutrition Coordinating Center, Minneapolis, Minn, USA) to complete the nutrient analysis [17]. The Dietwin® nutrition software (version 8.0) was used for the dietary quantification of the food recalls. To determine dietary intake, patients recorded the amount of ingested food in dietary diaries and the daily number of meals (breakfast, lunch, dinner and snacks, etc). Skilled dieticians trained patients how to record the total food intake in the diary by household measures, and also instructed them how to take the measures of the utensils before starting food record. The total dietary nutrients intake was examined, and the daily intake for each subject was calculated as the average of the three-day food records.

### Laboratory test

3-month average values of routine laboratory data were performed. Pre-hemodialysis blood samples were obtained. Serum albumin, serum transferrin, creatinine, urea nitrogen, hemoglobin were tested. Serum albumin level was determined by a bromocresol green dye binding colorimetric assay. Serum transferrin level was measured by the Ciba-Cornings Automated Chemiluminescence System (ACS180), employing a two-site chemiluminometric (sandwich) immunoassay, which uses constant amounts of two anti-ferritin antibodies. Creatinine and urea nitrogen were tested by TBA-120 auto-analyzer method while hemoglobin was measred by routine automated method.

### Nutrition and inflammation evaluation Scores

Nutritional and inflammatory scoring assessed by MIS was performed on the participating hemodialysis patients. MIS has 10 components (weight change, dietary intake, gastrointestinal symptoms, functional capacity, comorbidity, subcutaneous fat, muscle wasting, BMI, serum albumin level, and total iron-binding capacity or serum transferrin level) and each has four levels of severity, from 0 (normal) to 3 (very severe) [11]. The sum of all easy-to-assess scores of 10 MIS components ranges from 0 to 30, denoting the increased severity of malnutrition and inflammation.

### Statistical analysis

Descriptive statistics and regression analysis were performed with the statistical software package (SPSS17.0). The data that present a normal distribution were expressed with mean ± SD. If not, they were expressed as median (min-max). Differences of normal distributed continuous variables between groups were determined by unpaired t-test, while non-normal distributed continuous variables were compared by Mann-Whitney U-test. Categorical variables were analyzed by Chi-square test. Pearson's correlation was used to assess the strength of association between MIS scores. Operating characteristics curve (ROC) analysis was used to quantify the assessing value of independent parameters for nutrition and inflammatory status. A P-value of<0.05 was considered statistically significant.

## Results

### Clinical and dialysis characteristics

A total of 75 patients (51 men and 24 women) participated in this study. The mean age of the whole population was 62.75 ± 14.04 years old. The cause of end-stage renal disease was chronic glomerulonephritis (40 patients), diabetic nephropathy (20 patients), hypertensive nephropathy (8 patients), polycystic kidney disease (3 patients), and other etiologies (4 patients). Hemodialysis patients were maintained on their regular prescription, 2–3 times per week for 4–5 hours per session. The blood flow ranged from 230 to 300 mL/min, with a dialysate flow rate of 500–600 mL/min. The other clinical and dialysis characteristics were showed in table 1.

**Table 1:** Clinical and dialysis characteristics of the enrolled patients.

Characteristics	total enrolled patients (n = 75)
Age (years)	62.75±14.04
Sex ratio (Male/Female)	51/24
Number of comorbidities	
Chronic glomerulonephritis	40
diabetic nephropathy	20
Hypertensive nephropathy	8
polycystic kidney disease	3
other etiologies	4
SBP (mmHg)	156.36±2.03
DBP (mmHg)	97.27±1.95
Creatinine ( µmol/L)	906.27±64.39
BUN (mmol/L)	23.59±1.90
Albumin (g/L)	37.91±1.79
Hemoglobin (g/L)	107.55±3.48
Transferrin (g/L)	48.58±4.00

All values are expressed as mean ± SD.

Abbreviations: BUN, urea nitrogen; DBP, diastolic blood pressure; SBP, systolic blood pressure.

### Anthropometric assessments and the relationship with MIS

**Figure 1:**
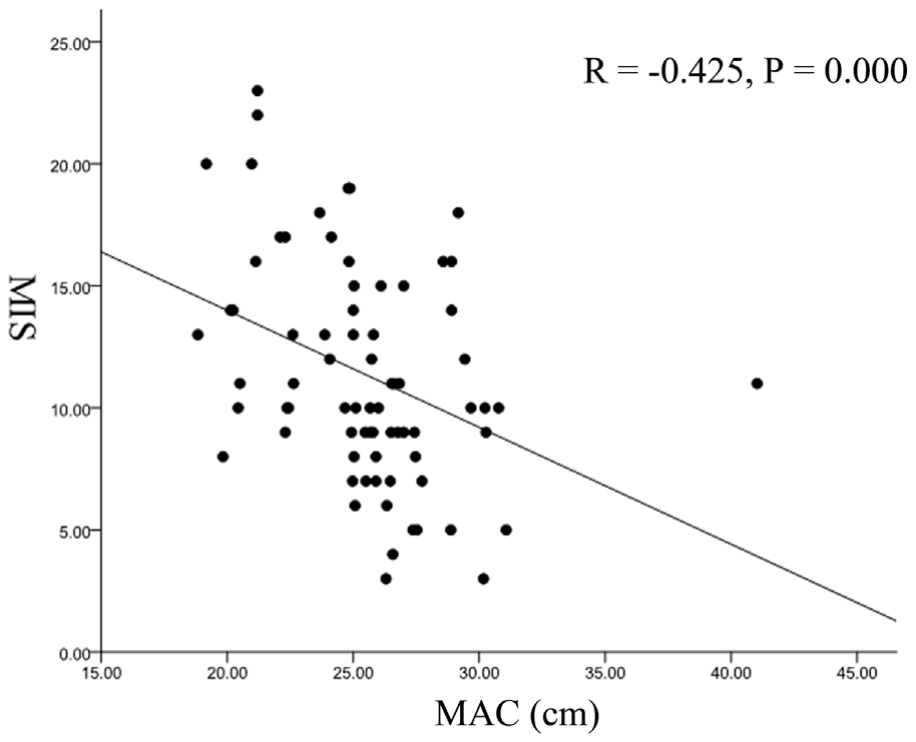
Scatter plots, regression line reflecting the correlation between mid arm circumference (MAC) and malnutrition-inflammation score (MIS).

**Figure 2:**
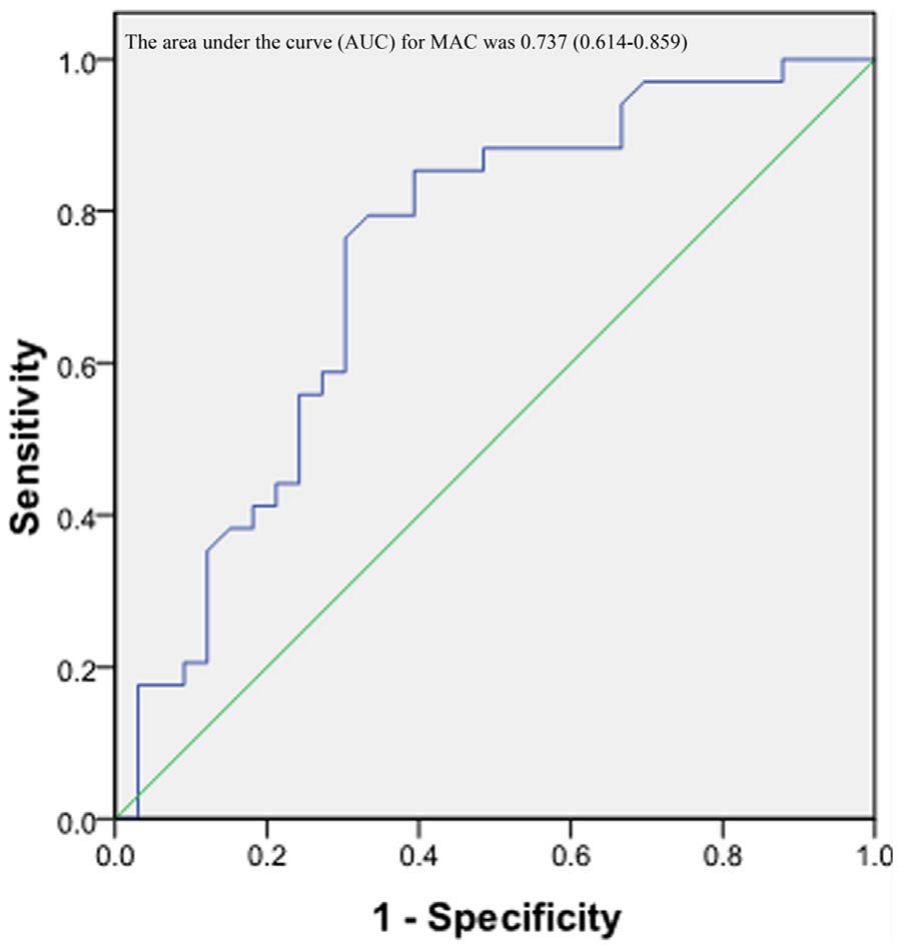
Receiver-operating characteristic (ROC) curve for mid arm circumference (MAC) in assessing nutritional and inflammatory status using malnutrition-inflammation score (MIS) as the reference standard (*P<0.05). For each screening test, sensitivity is plotted against 100-specificity. The ideal test would have 100% sensitivity and 100% specificity and reach the upper left corner of the graph; a test with no diagnosis value would lie along the diagonal between the lower left corner and the upper right corner.

In this study, a score of less than 10 is normal and more than 10 is considered malnourishment. Based on the MIS scoring method, two groups were classified: normal nutrition group (7.77 ± 2.15) and malnutrition group (15.12 ± 3.39) (Table 2). MAC reflects the skeletal mass and the mean value of MAC was 25.78 ± 2.09 cm. MAMC, measuring the protein status in the body was 22.43 ± 3.26 cm on average. We found that there was a significant difference of MAC between two nutritional groups (*P* = 0.012), while no significance of MAMC was found (*P* = 0.070) (Table 2). Then we try to confirm the above findings further through Pearson's correlation (Table 3). It was showed that both the anthropometric assessments of MAC and MAMC had significant negative correlations with MIS (MAC, r = −0.425, *P* = 0.000; MAMC, r = −0.334, *P* = 0.006), and positively correlated with BMI (MAC, r = 0.705, *P* = 0.000; MAMC, r = 0.526, *P* = 0.000). And it indicated that MAC was a good marker for nutritional and inflammatory status than MAMC although MAMC was also significantly associated MIS. Then the scatter plots, regression line and ROC curve analysis were used to analyze the above conclusion (Figure 1, 2). The area under the curve (AUC) for MAC was 0.737 (0.614–0.859) (*P*<0.05). MAC with a threshold value of 25.05 cm provided 76.5% sensitivity and 69.7% specificity.

**Table 2:** Anthropometric values classified with two groups according to malnutrition-inflammation score (MIS) in hemodialysis patients.

Variable	total	normal	malnutrition	P
	n = 75	n = 39	n = 36	
MAC (cm)	25.78 ± 2.09	26.47 ± 2.51	24.34 ± 4.08	0.012*
MAMC (cm)	22.43 ± 3.26	22.67 ± 1.86	21.45 ± 3.35	0.070
MIS	11.36 ± 4.52	7.77 ± 2.15	15.12 ± 3.39	0.000*

All values are expressed as mean ± SD.

* P<0.05, normal nutrition group *vs.* malnutrition group.

Abbreviations: MAC, mid arm circumference; MAMC, mid arm muscle circumference.

**Table 3:** MAC and MAMC were association with malnutrition-inflammation score (MIS) and body mass index (BMI) using Pearson's correlation.

Variable	MIS		BMI
	r P		r P
MAC (cm)	−0.425 0.000*		0.705 0.000*
MAMC (cm)	−0.334 0.006*		0.526 0.000*

r for Pearson correlation coefficients (*P<0.05).

Abbreviations: MAC, mid arm circumference; MAMC, mid arm muscle circumference.

### Dietary micronutrients intake in normal nutrition and malnutrition groups

The accurate dietary micronutrients intake was showed in table 4 and table 5. In this study, we mainly focus on the micronutrients not protein or energy dietary intake which showed no significant association with MIS (*P*>0.05, data not shown). Then we analyzed the micronutrients of the two nutritional groups according to MIS. It was found that micronutrients, such as Se, Copper (Cu), Iodine (I) and Manganese (Mn) intake maybe important early and alarming markers for the nutritional and inflammatory status. We showed that the median intake of Se, Cu, I and Mn in both groups (malnutrition group *vs.* normal nutrition group) were as follows: Se (44.70 (8.96–67.30)ug *vs.* 52.14 (18.32–163.53)ug, *P* = 0.002), Cu (1.19 (0.50–1.68)mg *vs*. 1.43 (0.78–3.95)mg, *P* = 0.021), I (1.13 (0.00–5.80)ug *vs.* 4.09 (0.00–46.60)ug, *P* = 0.016), Mn (3.39 (1.85–5.06)ug *vs*. 4.26 (1.69–8.95)ug, *P* = 0.003) (Table 4). In this study there were no apparent differences among the vitamins between the normal nutrtition group and malnutrition group (Table 5).

**Table 4:** Dietary minerals intake status classified by two groups according to MIS.

Variable	total	normal	malnutrition	P
	n = 75	n = 39	n = 36	
Ca (mg)	398.67 (120.00–1145.67)	406.50 (161.00–1145.67)	377.33 (120.00–985.33)	0.962
P (mg)	926.00 (212.00–1554.00)	950.00 (485.67–1554.00)	886.84 (212.00–1265.00)	0.146
K (mg)	1601.11 (478.67–3034.67)	1742.34 (598.00–3034.67)	1404.61 (478.67–2685.67)	0.488
Na (mg)	742.68 (146.57–2946.20)	795.60 (146.57–2946.20)	703.02 (260.57–1419.83)	0.588
Mg (g)	238.50 (87.67–625.33)	252.50 (145.00–625.33)	233.67 (87.67–334.33)	0.102
Fe (mg)	15.92 (7.10–34.17)	17.18 (9.20–34.17)	14.60 (7.10–25.33)	0.336
Zn (mg)	10.38 (3.89–16.83)	10.77 (5.50–16.83)	9.93 (3.89–14.64)	0.456
Se ( µg)	49.85 (8.96–163.53)	52.14 (18.32–163.53)	44.70 (8.96–67.30)	0.002*
Cu (mg)	1.34 (0.50–3.95)	1.43 (0.78–3.95)	1.19 (0.50–1.68)	0.021*
Mn (mg)	3.94 (1.69–8.95)	4.26 (1.69–8.95)	3.39 (1.85–5.06)	0.003*
Iodine ( µg)	1.10 (0.00–46.60)	4.09 (0.00–46.60)	1.13 (0.00–5.80)	0.016*

All values are expressed as median (min-max).

P value based on t-test for parametric continuous variables is used for comparison among the two groups: normal nutrition group and malnutrition group (*P<0.05).

Abbreviations: Ca, calcium; Cu, copper; Fe, iron; K, potassium; Mg, magnesium; MIS, malnutrition-inflammation score; Mn, manganese; Na, sodium; P, phosphorus; Se, selenium; Zn, zinc.

**Table 5:** Dietary vitamins intake status classified by two groups according to malnutrition-inflammation score (MIS).

Variable	total	normal	malnutrition	P
	n = 75	n = 39	n = 36	
Vitamin A ( µg)	476.50 (39.67–2374.67)	474.84 (39.67–1788.67)	476.50 (154.67–2374.67)	0.869
Thiamine (mg)	0.92 (0.19–1.70)	0.90 (0.40–1.70)	0.98 (0.19–1.60)	0.612
Riboflavin (mg)	0.87 (0.25–4.01)	0.88 (0.40–4.01)	0.72 (0.25–2.02)	0.928
Vitamin B6 (mg)	0.11(0.00–1.00)	0.11 (0.00–1.00)	0.13 (0.00–0.31)	0.246
Folic acid ( µg)	43.25 (0.00–162.10)	36.35 (0.00–162.10)	52.35 (0.00–148.10)	0.849
Vitamin B3(mg)	15.61 (4.13–29.50)	16.54 (6.83–29.50)	13.60 (4.13–24.17)	0.362
Vitamin C (mg)	87.67 (0.40–571.33)	93.42 (5.20–204.80)	84.12 (0.40–571.73)	0.165
Vitamin E (mg)	22.10 (5.31–40.24)	22.36 (5.31–40.24)	20.05 (8.78–31.03)	0.100

All values are expressed as median (min-max).

* P<0.05, normal nutrition group *vs.* malnutrition group.

## Discussion

Malnutrition can be assessed by different tools. Some biochemical parameters (e.g. serum albumin) and anthropometric measurements (e.g. body weight) are effective in identifying the patients with malnutrition, however, they have some limitations affected by some non- nutritional factors, such as edema, liver disease, and chronic inflammation, etc [18], [19]. BMI is also a traditional method to assess the nutritional status, but it is not a sensitive marker for malnutrition [20], [21]. Since malnutrition and inflammation are not recognizable by only one or two markers, it is advisable to use multiple markers. Currently, the evaluation of nutritional and inflammatory status is often overlooked and the assessment standard is not definite in Chinese hemodialysis patients. MAC is widely used in nutritional status assessment due to its relative effectiveness and association with the quality of life [22]. In this study, we found that MAC maybe a better marker for the nutritional and inflammatory status which was consistent with previous study. Janardhan et al. showed that MAC was significantly correlated with dialysis malnutrition score [23]. Furthermore, Stosovic et al. demonstrated that MAC was the most predictive anthropometric factor on mortality in hemodialysis patients [24]. However, some other studies did not find MAC has a relation with inflammation in hemodialysis patients [25], [26]. More studies are needed.

Since China is a developing country, the malnutrition is still a big problem in most regions. Meanwhile, the eating habit of Chinese population, whose three meals contain a lot of carbohydrate, is very different from westerners. So it is important to pay attention to the micronutrients intake especially in hemodialysis patients. A study by Slomowitz et al [27] showed a direct correlation between energy consumption and alterations in nutritional parameters. And a protein intake of 1.2 g/kg/day and a caloric intake of 35 kcal/kg/day is recommended to maintain a neutral nitrogen balance and prevent changes in body composition (K/DOQI, 2000) [28]. However, there are few guidelines on the micronutrients intake level, especially the trace minerals in China. In our study, a 3-day food record was used to estimate dietary nutrients intake of hemodialysis patients. Low dietary intake of micronutrients was observed with regard to Se, Cu, I and Mn among hemodialysis patients with malnutrition (Table 4). And it indicated that the less intake levels of Se, I, Cu and Mn, the worse malnutrition and inflammatory status would be. It is hypothesized that reduction of Se, I, Cu and Mn intake may be involved in the development of malnutrition and inflammation. In fact, previous studies have found that in peritoneal dialysis patients, lower micronutrient intakes (e.g. sodium, calcium, vitamins A and B2) were associated with malnutrition and inflammation [29]. However, we did not find significant difference of diversity of vitamins between normal nutrition group and malnutrition group. That may because the peritoneal dialysis patients have different characteristics comparing with hemodialysis patients. Moreover, evidence also suggests that there is high prevalence of trace elements deficiency in hemodialysis patients [6]. Some micronutrients deficiencies in hemodialysis patients may contribute to the development of atherosclerotic cardiovascular disease [6]. As we all know, Se is an essential micronutrient for humans. Se deficiency has been identified in China, and it results in thyroid injury and decreases thyroid hormone production. Moreover, it may cause Keshan's disease, an endemic cardiomyopathy, mainly affecting children and women of childbearing age. Recently, a randomized double-blind placebo-controlled trial found that Se supplementation improved the nutritional status of hemodialysis patients and decreased the MIS score and the levels of interleulin-6 [30]. Low serum Cu levels were also present in hemodialyisis patients [31]. Dietary Cu deficiency is involved in erythropoietin-resistant anemia in hemodialysis patients [32]. It was known that Cu has a role on hemoglobin synthesis and is a cofactor for Cu/Zn superoxide dismutase [33]. Guo et al. found that Cu/Zn rations were associated with nutritional status, oxidative stress, inflammation, and immune abnormalities in peritoneal dialysis patients [34]. However, in hemodiaysis patients, Guo et al. found the levels of Cu were increased which was contrary to our study and other studies [31], [35]. The possible explanation may be that the release of Cu was caused by inflammatory tissue damage. And our enrolled patients had a low levels of Cu may also attribute to the deficient intake of pluck or nuts. Little literature was reported on the I intake in the hemodialysis patients, but there was deficiency in I intake in renal translation patients [36]. In hemodialysis patients, the reduction of I may attribute to the thyroid dysfunction. Previous study found that chronic renal failure affected thyroid function and the storage of I was increased which induced a decrease of serum I [37]. Mn is also an essential trace element that is required for the activity of several enzymes such as metalloenzyme superoxide dismutase (SOD) which is important for resisting excess oxidative stress in hemodialysis patients. Less than 5% of Mn is absorbed in small intestine. And Mn absorption is possibly decreased in iron deficiency which providing a rationale for Mn deficiency in hemodialysis patients (who are often iron deficient) [38], [39]. In addition, high dietary intake of fiber can also decrease the absorption of Mn [39] which may be one cause for Chinese hemodialysis patients (whose three meals containing abundant dietary fiber).

This study disclosed some nutritional problems usually overlooked in hemodialysis patients. And the results here emphasized the importance of micronutrients supplementation. However, more work is needed to define acceptable standard for the relevant micronutrients intake of hemodialysis population. In clinic, diverse types of dietary restrictions are imposed on hemodialysis patients, such as restricting phosphorus or potassium intake, some of which may cause more harm than benefit. Since malnutrition increases morbidity and mortality, and some of these effects resulting from specific micronutrient deficiencies. Hence, dietary micronutrients assessment is of paramount importance in assessing the nutritional and inflammatory status and providing optimal management among the hemodialysis patients.

Although we found micronutrients supplementation were important for hemodialysis patients, there are several limitations in this study. First, we only measured the dietary micronutrients. The serum levels of the micronutrients such as serum calcium, phosphorus, etc which may be important for nutritional status in hemodialysis patients were not tested. Second, some hormones e.g. PTH which may indirectly influence the absorption of micronutrients, were also not measured. Furthermore, the sample size is relatively small, and more large-scale clinic studies are needed.

In conclusion, MAC may be an appropriate and better indicator to assess the nutritional and inflammatory status. Dietary intake was generally characterized by the less intakes of Se, Cu, I and Mn in South China malnutrition hemodialysis population. Dietary intake of Se, Cu, I, and Mn level may be alarming and early markers to notice the nutritional and inflammatory status and notify the potential malnutrition-inflammation-atherosclerosis syndrome. Our results ask for an imperative re-evaluation of the dietary micronutrients intake requirements in hemodiaysis patients. Larger samples and more related studies are needed to verify our findings in hemodialysis population.
